# DMCA-GAN: Dual Multilevel Constrained Attention GAN for MRI-Based Hippocampus Segmentation

**DOI:** 10.1007/s10278-023-00854-5

**Published:** 2023-09-21

**Authors:** Xue Chen, Yanjun Peng, Dapeng Li, Jindong Sun

**Affiliations:** 1 College of Computer Science and Engineering, Shandong University of Science and Technology, Qingdao, 266590 Shandong China; 2 Shandong Province Key Laboratory of Wisdom Mining Information Technology, Shandong University of Science and Technology, Qingdao, 266590 Shandong China

**Keywords:** Hippocampus segmentation, Dual generative adversarial network, Attention mechanism, Information constraint, Magnetic resonance images

## Abstract

Precise segmentation of the hippocampus is essential for various human brain activity and neurological disorder studies. To overcome the small size of the hippocampus and the low contrast of MR images, a dual multilevel constrained attention GAN for MRI-based hippocampus segmentation is proposed in this paper, which is used to provide a relatively effective balance between suppressing noise interference and enhancing feature learning. First, we design the dual-GAN backbone to effectively compensate for the spatial information damage caused by multiple pooling operations in the feature generation stage. Specifically, dual-GAN performs joint adversarial learning on the multiscale feature maps at the end of the generator, which yields an average Dice coefficient (DSC) gain of 5.95% over the baseline. Next, to suppress MRI high-frequency noise interference, a multilayer information constraint unit is introduced before feature decoding, which improves the sensitivity of the decoder to forecast features by 5.39% and effectively alleviates the network overfitting problem. Then, to refine the boundary segmentation effects, we construct a multiscale feature attention restraint mechanism, which forces the network to concentrate more on effective multiscale details, thus improving the robustness. Furthermore, the dual discriminators D1 and D2 also effectively prevent the negative migration phenomenon. The proposed DMCA-GAN obtained a DSC of 90.53% on the Medical Segmentation Decathlon (MSD) dataset with tenfold cross-validation, which is superior to the backbone by 3.78%.

## Introduction

As an essential component of the brain central nervous system, the hippocampus controls memory storage and cognitive learning and is the crucial decision-maker for spatial orientation and response inhibition [1]. However, it is highly susceptible to damage due to hypoxia, anemia, hypoglycemia, and encephalitis. Therefore, the volume and morphological changes in the hippocampus provide essential guidance in diagnosing and treating neurological diseases.

For example, in Alzheimer’s disease (AD) [2], the hippocampus is the earliest region to be damaged. Precise measures of atrophy extent can predict the stages of dementia. For early-stage patients in the mild cognitive impairment (MCI) [3] stage, timely diagnosis and early psychological treatment can delay or even prevent their deterioration to AD. In temporal lobe epilepsy [4], schizophrenia [5], depression [6], and posttraumatic stress disorder (PTSD) [7], patients also have varying degrees of hippocampal atrophy. Among them, head atrophy of the hippocampus in schizophrenia is evident and more serious in the left; tail atrophy is obvious and irreversible in severe depression due to the excessive glucocorticoids released by long-term mental pressure; and temporal lobe epilepsy further shows sclerosis or vessel rupture within the hippocampus. In contrast, the hippocampal volume can increase through aerobic exercise in healthy individuals, especially adults aged 55 to 80. This improves their spatial memory capacity [8]. Therefore, the morphological features of the hippocampus are crucial. The primary need is to precisely segment the hippocampus from MR images. Manual segmentation remains the gold standard for clinical applications, which is time-consuming and susceptible to differences in empirical knowledge among experts. As a result, an accurate automatic hippocampus segmentation method is of significant clinical relevance.

Magnetic resonance imaging (MRI) [9] is an important clinical technique for monitoring subtle variations in hippocampal structure. In particular, the high-contrast image obtained by the T1w sequence provides significant advantages in hippocampal volume assessment. T2w and T2-FLAIR sequences can ensure the imaging signal-to-noise ratio and display the internal structure of the hippocampus. However, since both the hippocampus and its amygdala are gray matter structures, their boundaries are blurry and highly confusing in MR images. In addition, the hippocampus accounts for a small proportion of brain imaging, with an irregular shape and high individual variability. In recent years, automatic MRI-based hippocampal segmentation has been a challenging topic in the medical field, and the major difficulties are as follows:

1. The complex structure of the hippocampus in MR images results in unclear boundaries and easy confusion with surrounding tissues;
2. The small proportion of the hippocampus causes a serious class imbalance between foreground and background voxels;
3. The high resolution of MRI images with significant amounts of noise results in numerous irrelevant features interfering with the segmentation accuracy.

In this paper, focusing on the exact clinical task of precise hippocampus segmentation in high-frequency MR images, we proposed a dual multilevel constrained attention GAN (DMCA-GAN) for MRI-based hippocampus segmentation. To better show the volume, we adopt T1w hippocampal MRIs, which are all from the 2018 Medical Segmentation Decathlon (MSD) challenge [10, 11]. First, we applied random preprocessing operations to minimize noise interference, increase the proportion of foreground information, and prevent overfitting. Then, we evaluated the performance of our designed dual discriminator generative adversarial network in capturing global and local information of the MRI-based hippocampus. In addition, considering the high-frequency noise issue in MRI, we constructed an information entropy constraint unit ($I_{FC}$) and a multilevel feature extraction mechanism (*MFCM*) and demonstrated their capabilities through a series of ablation experiments. Finally, we compared our proposal with previous studies and achieved an outstanding outcome.

## Related Works

Over the last decade, dominant algorithms of hippocampal segmentation have mainly been based on multiatlas segmentation (MAS) [12] and deep learning (DL) [13]. In this section, a brief and clear discussion of the most relevant works is presented. A quantitative overview of previous work is presented in Table 1.

### Prior Hippocampus Studies Based on MAS

MAS generally utilizes existing expert prior knowledge and atlas clustering to obtain the registration between atlas images and target domain images, thus achieving segmentation in the target domain. The crucial technique is patch alignment and label fusion, typically applying multiatlas pixel-by-pixel alignment and label voting majority voting methods, which is currently popular research.

To overcome the limitations of the small available dataset and the manual labeling gold standard, Thyreau et al. [14] created various synthetic data by the software package FreeSurferto and flipped all right hippocampi to the left before rigid registration, thus simplifying the network to learn only the left side. Their DSC average exceeded 0.83. However, alignment errors are unavoidable, and the possible clinical anatomical variations in the hippocampus are impossible to simulate.

By MAS-based longitudinal label estimation, Guo et al. [15] used the output estimation of the upper phase as the temporal context features to guide the current phase appearance feature extraction. Moreover, they refined boundaries by the designed longitudinal CRRF (L-CRRF) algorithm. They exploited well the knowledge of a priori markers of hippocampal development. However, their dataset contained only 10 subjects, and the average DSC was merely 65.35%.

In addition, Wu et al. [16] creatively combined features of MRI (T1 and T2) images with resting-state fMRI (rs-fMRI). Training by random forest, they achieved a mean DSC of 0.69 for eight healthy subjects [17]. Their method demonstrated multimodal fusion to enhance segmentation performance. However, due to the limited available data (particularly poor rs-fMRI data) and incomplete learning strategy of association relations, the results tend to fall into local optima rather than global optima. At present, the mainstream segmentation task is still to measure the degree of whole atrophy.

Although MAS-based methods for hippocampus segmentation currently perform better, their accuracy is usually limited to single specific tasks only. In addition, training them requires large amounts of manually labeled features such as textures and boundaries. Compared with them, novel DL methods have been proven to achieve more general and efficient performance for segmentation tasks.

### Prior Studies Based on DL

Deep learning, as a maturing end-to-end method, is gaining traction in the semantic segmentation of medical images. Over the past decade, with the development of hardware support for processing units and parallel technology, CNNs have performed excellently in pixel-level learning representation of medical images [18–25]. Satisfactory results have been achieved in glaucoma [26], brain tumors [27, 28], prostate [29], skin lesions [30], heart [31] and other parts, especially in the hippocampus. It realizes the most advanced segmentation performance available, which enables automatic data-driven learning of hippocampus features.

Aimed at multitask joint training to promote hippocampus segmentation, Liu et al. [32] built a multitask deep CNN model. They implemented hippocampal segmentation and AD classification on the Alzheimer’s Disease Neuroimaging Initiative (ADNI) [33] database. For the segmentation task, a DSC of 87.0% was achieved. Although they believe that multimodel outperforms single-model methods, training a deeper CNN is more time-consuming and resource intensive. The current public hippocampus datasets are small and insufficient to learn precise foreground information within the base CNN. In addition, the robustness of high-dimensional MRI data during CNN training and the overfitting caused by the fixed 3D patch have yet to be improved.

Hazarika et al. [34] aimed to improve the efficiency of the basic 2D U-Net model. They revisited and tweaked the original framework of U-Net by replacing all kernels of sizes 3$\times$3 with three optional 1$\times$1, 3$\times$3, and 5$\times$5 kernels. Their innovation was well applied in 2D MRI-based hippocampus segmentation, obtaining an average DSC of 96.5%. However, trading higher convolutional parameters for better performance inevitably increases the computational cost, and it limits model generalization to applications. In the clinical case, for instance, hippocampal scans are often 3D MR imaging, while the model is insufficient to process these 3D data.

Moreover, to alleviate the catastrophic forgetting phenomenon in segmentation models, Ranem et al. [35] combined the recent popular Vision Transformer (ViT) [36] with nnU-Net [37] and achieved an 89.8% DSC value on MSD. However, due to the large resources needed for nnU-Net, the optimization algorithm remains to be explored. As the author mentioned, it could consider replacing the convolutional layer with convolutional attention or the ViT layer with a batch normalization layer. Additionally, the negative impact of ViT’s self-attention mechanism over time has yet to be solved.

In addition, collaborative networks can also enhance the segmentation accuracy of small-size datasets, such as Md-Unet designed by Lin et al. [38]. They attempted to extract shared features from multiple datasets simultaneously, thus helping to balance multidomain segmentation performance. Compared to its baseline 3D $U^{2}Net$ average DSC of 70.2%, Md-Unet is 3.7% higher. However, multiple dataset segmentation still requires improvement in minimizing imbalance among datasets and class imbalance in each dataset. For instance, Md-Net achieved a segmentation DSC of 91.9% for the binary heart but only 69.9% for the three-classified hippocampus (anterior is 64.0%, while posterior is 75.7%).

More recently, deep generative and adversarial networks (GAN) have achieved satisfactory performance on feature representations [39–43]. The GAN-based segmentation network designed by Shi et al. [44] well-achieved the smoothness of edge and the spatial consistency of segmentation. In particular, their constructed generator, namely UG-Net, was the modification of U-Net. By alternately training the UG-Net and the regular discriminator D, they obtained decent pixel-level segmentation results for seven subfields of average DSC of 85.2%. But their segmentation strategy does not perform well in small subfields because of the few corresponding sample voxels. Following the same idea, Chen et al. [45] used 3D CNN as a generator and SVM as a discriminator, which obtained average DSC of 96.5%. Although SVM is more effective for small medical image segmentation, the increased complexity of the hybrid model is considerable. Moreover, [46, 47] used the hippocampal segmentation results of semisupervised GAN networks for early Alzheimer’s disease detection, which similarly obtained excellent detection results.

### Improvement Mechanisms on Segmentation Networks

Since the attention mechanism can automatically concentrate on salient and inhibit irrelevant features, it has gradually been applied in DL models. To compensate for the segmentation loss caused by the complex structure of the pancreas, Li et al. [48] established a DCNN-based multiscale selection unit, namely, MSC-DUnet, which captured global spatial features and multilayer local features from multiple receiver fields. The authors claimed that their segmentation network was superior to the baseline of 5.1% in DSC. Luo et al. [49] furnished a GAN with a sense-aware information bottleneck (SIB), thus simplifying feature alignment and stabilizing the adversarial training process. To enhance the degree of feature alignment between the origin and target domains of GAN, Luo et al. [50] further increased the weight of those poorly aligned adversarial losses through a collaborative training approach. Both improvements gained competitive accuracy compared with state-of-the-art unsupervised domain adaptation (UDA) approaches.

**Table 1:** Qualitative comparison of prior hippocampus segmentation studies

**Key Method**	**Dataset**	**Modalities**	**Training Strategies**	**Average Dice(%)**	**Year**
software data synthesis and multiple cohorts transferring with 3D CNN [14]	ADNI(135), HCP(547), AOBA(317), TRGY(112), OASIS(58), ABIDE(197)	3D T1 MRI	3D volumes (48$\times$72$\times$64)	$DSC_{ADNI}$=85 $DSC_{HCP}$=83 (left/right=82/84)	2018
L-CRRF with dense CRFs [15]	10 infant brain from UNCCH	3D T1w MRI (192$\times$156$\times$144)	3D volumes (13$\times$13$\times$13)	$DSC_{avg}$=65.3 $DSC_{2-week}$=58.8 $DSC_{3-month}$=63.6 $DSC_{6-month}$=66.1 $DSC_{9-month}$=68.0 $DSC_{12-month}$=67.0	2020
structured random forest with auto-context model [16]	8 private healthy subjects and 4 healthy subjects randomly from HCP	3T MRI(T1,T2), rs-fMRI	3D volumes (11$\times$11$\times$11)	$DSC_{avg}$=69.0	2018
multimodel deep CNN jointly with 3D DenseNet [32]	ADNI(449)	3D T1w MRI	3D volumes (64$\times$48$\times$64)	DSC=87.0	2020
Tweaked U-Net with three alternative kernels of sizes 1$\times$1, 3$\times$3, and 5$\times$5 [34]	ADNI (210)	3D T1w MRI	2D slices (256$\times$256$\times$1)	DSC=96.5	2022
nnU-Net with ViT [35]	HarP(270), Dryad(50), DecathHip(260)	3D T1w MRI	2D slices	$DSC_{DecathHip}$ =89.8 $DSC_{HarP}$=+4.8 $DSC_{dryad}$=+16.2	2022
GAN with modified U-Net generator [44]	Brain images from CIND center (MCI: 4, AD: 7, normal: 21)	3D T1w MRI, 3D T2w MRI	2D slices (128$\times$128)	$DSC_{avg}$=85.2	2019
dual-branch with improved SSA adapters [38]	MSD (Heart:20, Hippocampus:260, Pancreas:281, Spleen:41)	MRI, CT	2D slices, 3D patch	$DSC_{heart}$=91.9 $DSC_{hipp}$=69.9 $DSC_{pancreas}$=74.5 $DSC_{spleen}$=83.7	2022
GAN with 3D CNN generator and SVM discriminator [45]	Brain images from CIND center (MCI: 4, AD: 7, normal: 21)	3D T1w MRI, 3D T2w MRI	3D patch (128$\times$128$\times$128)	$DSC_{avg}$=96.5	2020

## Material and Methods

### Overview of the Proposed DMCA-GAN

**Fig. 1:**
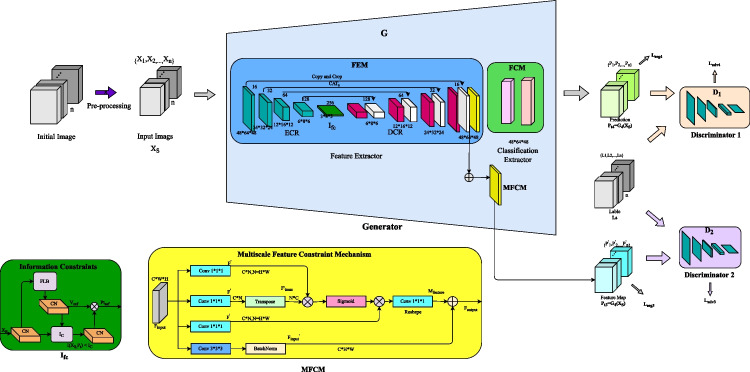
The proposed DMCA-GAN framework. In generative adversarial learning processing, discriminators D1 and D2 separately concentrate their attention on distinguishing feature maps and the ground truth under different receptive fields, equivalent to generating dual constraint mechanisms for G. This means that during feature extraction, G must generate sufficient effects on each decoding layer to trick both D1 and D2

Based on a basic generative adversarial network, the proposed dual-GAN exploits multilayer information constraints and context attention to affect multilevel perception. Figure 1 shows the basic framework of our proposed DMCA-GAN. For the raw data, preprocessing algorithms were performed before training, processing it into $X_{S} = \{X_{1},X_{2},...,X_{n}\}$, where *n* is the number of channels and $X_{i}$ is the 3D patch around the hippocampus. Later, $X_{S}$ is fed into generator *G* and trained up to two different multilevel feature vectors, denoted as $P_{t4} = \{P_{1},P_{2},...,P_{n}\}$ and $P_{t3} = \{P_{1}^{'},P_{2}^{'},...,P_{n}^{'}\}$, respectively. After that, $P_{t3}$ and $P_{t4}$ are sent into discriminators $D_{2}$ and $D_{1}$, respectively, to calculate the adversarial loss, jointly driving G trains more aggressively. More specifically, to enhance the effective representation of multiscale features in *G*, we explore bottleneck-based information constraints and cross-channel attention mechanisms. Our training goal is to balance the losses of the generative and adversarial models, thus making the generative model optimal for hippocampal segmentation. In addition, postprocessing optimizes the segmentation effectiveness.

### Preprocessing

**Fig. 2:**
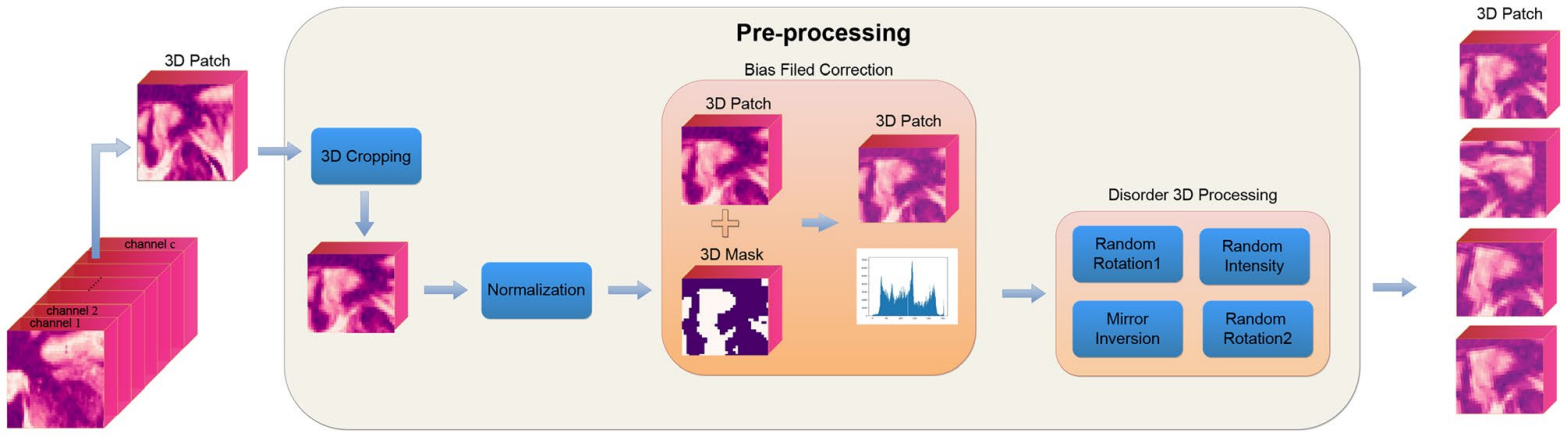
Preprocessing of hippocampal MR images. To facilitate visual observation, we present them in different shades of color. Parameter randomization is performed independently for each extracted 3D patch. In disordered 3D processing, random morphological microdithering is carried out to reduce the sensitivity of the training model to a single image and improve the robustness and generalization ability of our model

Considering the small quantity of data, the possible correlations between left and right tissues scanned from the same subject, and the different tissue contrasts and resolutions among different scans, we performed randomized data preprocessing before training. Referring to Fig. 2, there are five operations in our method: non-ROI pixel-level region cropping, patch sampling, normalization, bias field correction and fourfold data augmentation.

Regarding cropping and patch sampling, our methods are based on volumes. The optimal 3D patch size in the proposed network is 48$\times$64$\times$48, with the corresponding ablation experiments described in the “Ablation Studies” section. Specifically, we take the center coordinates of the point of the smallest data space, which contains the complete hippocampal structure, map it to all target spaces, and then crop them into the patch size of 48$\times$64$\times$48 with these coordinates as the center point. Since we applied four maximum pooling operations in the downsampling stage, the size of the cropped image in each dimension must be divisible by $2^4$.

In addition, considering the poor proportion of the hippocampus in MR images of the brain, to avoid loss drop volatility during training caused by no hippocampus within the extraction patches, we force at least 1/3 of the samples in each batch to contain prospects. Moreover, since the data volume and image size of the hippocampus are not large, we choose to process the data at the beginning of each training epoch rather than before entering the training network. This operation enhances the randomness and diversity of training data and causes only a small memory and time consumption increase. This randomization gives the network a strong generalization ability, which is beneficial for improving the accuracy and sensitivity of segmentation and preventing overfitting.

To accelerate the convergence of the network during training, we normalize patches with different intensities by performing z score normalization [51] for each sequence. It standardizes training data into the same order of magnitude and distribution as:

1 $$\begin{aligned} Z = \frac{x - \mu }{\sigma } \end{aligned}$$

where *x* is the pixel vector of the current 3D patch, $\sigma$ is the standard deviation of the current sequence at the pixel level, and $\mu$ is its mean.

**Fig. 3:**
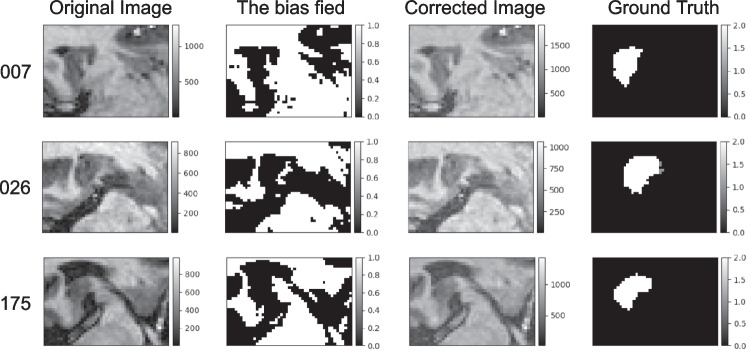
Three example sagittal diagrams of bias field correction on the hippocampus image. Each line from left to right is the current image number, the original image, the calculated bias, the corrected image and its corresponding label

More generally, at times, the same tissue may show different intensities due to image quality problems in MR images. It can mislead a segmentation model into learning them into two completely opposite groups. Usually, it is caused by magnetic fields and signal interference during scanning. To alleviate this problem, we refer to the Otsu algorithm [52] to perform N4 bias field correction on images [53]. As shown in Fig. 3, locally varying intensity tends to be smooth after correction, and the grayscale values of most of the background are reduced, which is beneficial to the extraction and learning of foreground information.

For data augmentation, we randomly selected 95% of the data for the following three operations on the x-, y- and z-axes. Random mirroring inversion with a probability of 0.5, random rotation with an angle between $[-15, 15]$ random and random intensity enhancement in the range of [$-$0.1$\times$ $\sigma$/2, 0.1$\times$ $\sigma$/2].

### DMCA-GAN

The generator G contains a segmented network as a feature extractor module (FEM) and a group of convolution blocks as a feature classification module (FCM). In the FEM, a U-shaped encoder-decoder structure is employed accomplish pixel-to-pixel multiscale feature prediction. Particularly, at the end of the encoder, a designed information constraint layer (ICL) is inserted to preserve the encoding of effective information, thus balancing the feature compression capability of the initial images with the feature representation capability of the label images. The decoder consists of two multiscale feature capture modules (MFCMs). It is a multiscale attention module with a pyramid-based pooling operation, facilitates capturing cross-scale features and enhances the correlation between pixels. Such effective preservation of valid information promotes FEM attention to target regions and contributes to improvement of FCM classification results.

Then, two discriminators D1 and D2 are introduced for adversarial learning. They receive the multilayer feature maps generated by G. Then, the generated maps are distinguished from the real label maps. Following this, they transmit the adversarial loss back to G, thereby facilitating G to generate closer feature maps to the ground truth, thus improving the segmentation performance. The designed joint loss function is described below.

For the input images $X_{S} = \{X_{1},X_{2},...,X_{n}\}$, its corresponding label is $L_{S} = \{L_{1},L_{2},...,L_{n}\}$, where *n* is the number of input patches. *G* learns a coding pattern $P_{t}$ under dual constraints of $D_{1}$ and $D_{2}$, and the two-scale level outputs generated by $P_{t}$ are $P_{t4} = G_{4}(X_{s})$ and $P_{t3} = G_{3}(X_{s})$, respectively. Our training goal is to learn a *P*, which is similar to $L_{s}$. The formula is expressed as follows.

2 $$\begin{aligned} Goal(\xi )=\max _{\xi }{I(P_{t},L_{s};\xi )}, s.t.I(P_{t},L_{s};\xi ) \le I_{C} \end{aligned}$$

where $\xi$ is the network parameter groups corresponding to optimal segmentation performance, $I(P_{t},L_{s};\xi )$ is the information correlation of $P_{t}$ and $L_{s}$, under $\xi$, i.e., $I_{c}$ is the output of *ICL* we defined.

#### Multilevel Information Constraints and Cross-Channel Attention Generator

When $X_{s}$ enters *G*, encoding layers of *FEM* ($EI_{s}$) first encode it, and then the decoding process ($DF_{s}$) decodes to obtain corresponding feature maps of $EI_{s}$. Nevertheless, EIs and DFs are concatenated via skip connections ($CAT_{s}$), which contributes to recovering downsampling information loss. The whole pixel-to-pixel segmentation can be denoted as:

3 $$\begin{aligned} \begin{aligned}&EI1(X_{si}) + EI2(X_{si}) + EI3(X_{si}) + EI4(X_{si}) = ECR(X_{si}); \\&DF1(X_{si}) + DF2(X_{si}) + DF3(X_{si}) + DF4(X_{si}) = DCR(X_{si}); \\&DF_{j}(X_{si}) = CAT(EI_{j}(X_{si}), DF_{j-1}(X_{si}));\\&i={1,2,...,n}, j = {1,2,3,4} \end{aligned} \end{aligned}$$

where *ECR* is the encoder of *FEM*, which has four convolution blocks and three max-pooling layers. Each convolution block contains convolution with filter sizes $3\times 3\times 3$ and step sizes of $1\times 1\times 1$, batch normalization (BN), and rectified linear unit activation functions (ReLUs). A max-pooling layers with filter sizes of $3\times 3\times 3$ and step sizes of $1\times 1\times 1$ is used to generate feature maps with a halved scale of $24\times 32\times 24$, $12\times 16\times 12$, $6\times 8\times 6$ and $3\times 4\times 3$. *DCR* is the decoder of *FEM*, which obtains the multiscale pixel-to-pixel feature maps corresponding to *FEM*.

Subsequently, the output layer, *FCM* consists of two convolutions with filter sizes of $3\times 3\times 3$ and $1\times 1\times 1$, and it modifies fitting results to obtain the final prediction. In particular, we add *ICL* to the last encoding layer of *ECR*, while $MFCM_{s}$ are in the last two decoding layers of *DCR*.

##### Efficient Information Constraints Based on Bottlenecks

Since the specific value of $I(P_{t},L_{s};\xi )$ cannot be calculated, a precise value of $I_{c}$ cannot be determined. Inspired by the bottleneck, we construct a Gaussian distribution (*q*(*y*)) based data ceiling constraint unit on the last encoder layer to extract significant information related to the target task in the feature vector. By introducing the Lagrange coefficient $\beta$, $Goal (\xi )$ is equivalently expressed as:

4 $$\begin{aligned} Goal(\xi ) = I(P_{t},L_{s};\xi ) - \beta I(P_{t},L_{s};\xi ) \end{aligned}$$

5 $$\begin{aligned} I(P_{t},L_{s};\xi ) = \iint dy dx p(y,x) \log \frac{p(y,x)}{p(y)p(x)} \end{aligned}$$

6 $$\begin{aligned} = \iint dy dx p(y\mid x)p(x) \log \frac{p(y\mid x)}{q(x)} + \int dy p(y)\log \frac{q(y)}{p(y)} \end{aligned}$$

7 $$\begin{aligned} y \in P_{t}, P_{t}=G(X_{S}), x \in X_{S} \end{aligned}$$

The upper bound constraints $I_{c}$ can be calculated by the following formula:

8 $$\begin{aligned} \begin{aligned} I_{C}&= \int p(x) CL[p(y\mid x)||p(x)]dx \\&= E_{x\sim {p(x)}}CL[p(y\mid x)||q(y)] \end{aligned} \end{aligned}$$

Accordingly, $I(P_{t},L_{S}) \le E_{x\sim {p(x)}}CL[p(y\mid x)||q(y)]$, and *CL* is the information constraint loss generated by the illustration of information constraints unit ($I_{fc}$).

**Fig. 4:**
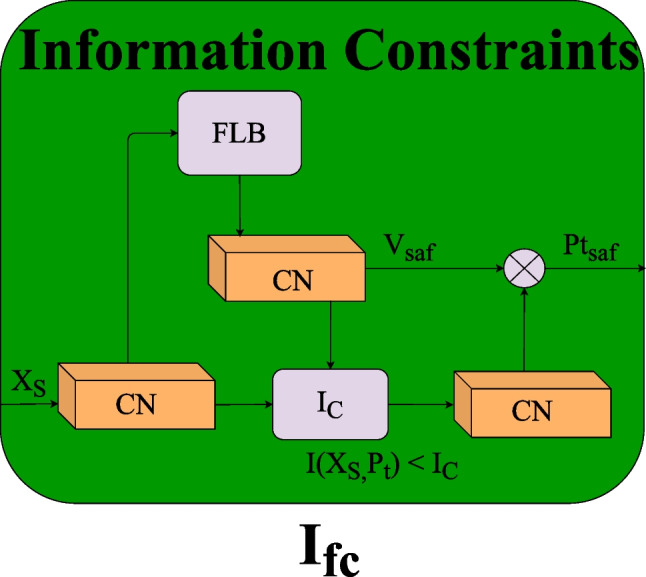
$$I_{fc}$$ in generator *G*

Using the upper bound vector $I_{C}$, we design a feature constrained unit ($I_{fc}$) to remove the noise in the inputs unrelated to segmentation. As illustrated in Fig. 4, *FLB* is a convolutional group composed of a $3\times 3\times 3$ convolution, a *ReLU* function, and a sigmoid activation function. It extracts salient features of the current channel feature map ($V_{saf}$) and is adaptively constructed to calculate the information constraint loss (*CL*). The feature weight formula ($Pt_{saf}$) is calculated as:

9 $$\begin{aligned} Pt_{saf}(X_{si},P_{ti}) = [(1-V_{saf})\bigodot I(X_{si},P_{ti})] \le I_{C} \end{aligned}$$

where $\bigodot$ is the corresponding channel multiplication operation.

Then, this constrained feature vector is fed into *FCM* to calculate the cross-entropy loss of segmentation and simultaneously calculate the adversarial loss between $P_{t}$ and $L_{s}$.

##### Multiscale Spatial Attention Feature Normalization Block (MFCM)

**Fig. 5:**
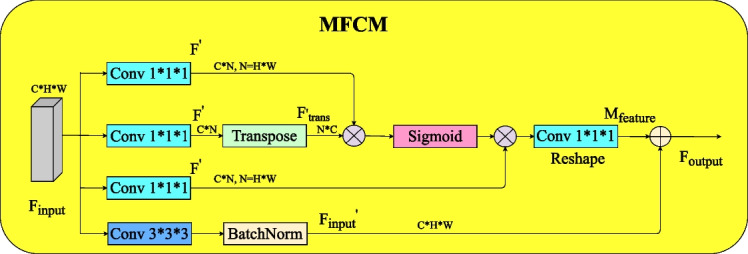
*MFCM* unit

The specific structures are illustrated in Fig. 5. Consider an aggregated input feature $F_{input} \in R^{C\times H\times W}$ of the *MFCM*, where *C*, *H* and *W* are the channel, height, and width of the input, respectively. $F_{input}$ is first reshaped to $F^{'} \in R^{C\times N}$, $N=H\times W$ by a $1\times 1\times 1$ convolution operation and transposed to $F_{trans}^{'} \in R^{N\times C}$. Subsequently we multiply $F^{'} \in R^{C\times N}$ and $F_{trans}^{'} \in R^{N\times C}$. Preliminary attention feature maps are then generated by utilizing the softmax function. To obtain the final attention map, we multiply it and $F_{trans}^{'}$ and reshape the result to $M_{feature} \in R^{C\times H\times W}$ by a $1\times 1\times 1$ convolution. Eventually, the spatial attention features are normalized by $F_{output}=\delta \sum _{i=1}^{N}(M_{feature} \times F_{input}) + F_{input}^{'}$, where $F_{input}^{'}$ is the $F_{input}$ after $3\times 3\times 3$ convolution operations for refinement.

**Fig. 6:**
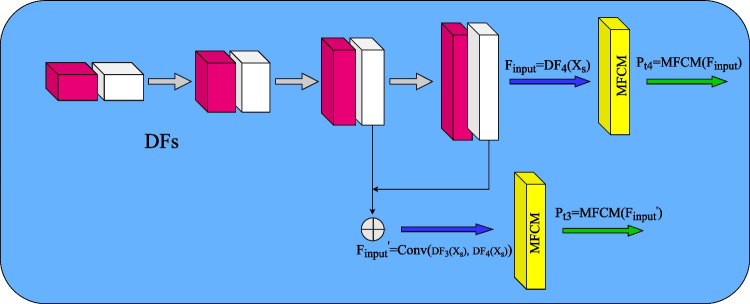
*MFCM* unit in the terminal output of *FEM* in generator *G*

To eliminate the interference of excessive noise and increase the concentration of useful pathological features in the multiscale decoding layer, we add the MFCM to the feature maps of the last and last two decoding layers, as depicted in Fig. 6.

#### Dual Adversarial Network for Multiscale Feature Discrimination

$D_{1}$ and $D_{2}$ utilize 5 identical convolution layer constructions, with corresponding kernel sizes of $7\times 7\times 7$, $5\times 5\times 5$, $3\times 3\times 3$, $3\times 3\times 3$ and $3\times 3\times 3$, in stride steps of 2. Both discriminators serve in determining whether the inputs are the predicted maps generated by $G (y \in P_{t}, p_{t}=G(X_{S}))$ or real label images $L_{S} (z \in L_{S})$. As shown in Fig. 1, the generated images are $P_{t4} = G_{4}(X_{s})$ and $P_{t3} = G_{3}(X_{s})$. Their task is to distinguish *y* from *z* as much as possible and output the probability values of the judgments. $D_{1}$ and $D_{2}$ pass these adversarial losses back to *G*, forcing *G* to segment more realistic segmentation results to deceive $D_{1}$ and $D_{2}$. They are trained alternately, and as the adversarial loss converges to approximately 0.5, it almost achieves equilibrium. At this point, we consider the network segmentation performance optimal.

#### Loss Function

Our DMCA-GAN contains three main types of loss functions: segmentation loss in *G*, information *CL* in $I_{fc}$, and adversarial loss in $D_{1}$ and $D_{2}$.

##### Segmentation Loss in G

The segmentation loss calculates the discrepancy between the predictions generated by G and its corresponding pixel-level ground truth, which can be formulated as:

10 $$\begin{aligned} L_{seg}(G) = E[l(G(X_{S}), L_{S})] \le I_{C} \end{aligned}$$

where *l*(.) is the appropriate loss function, *E*[.] is its vector-level mathematical expectations. Due to the deep imbalance between hippocampal and nonhippocampal pixels in MR images, the predictions still favor more dominant background pixels after preprocessing. Accordingly, here, we select the weighted sum of the binary cross-entropy loss and the Dice loss as the coefficient of the total segmentation loss metric.

11 $$\begin{aligned} L_{seg} = \sigma _{1}L_{BCE}(P_{t},L_{S}) + \sigma _{2}L_{Dice}(P_{t},L_{S}) \end{aligned}$$

12 $$\begin{aligned} L_{BCE}(P_{t},L_{S}) = -\beta L_{si}\log P_{ti} - (1-\beta )(1-L_{Si})\log (1-P_{ti}) \end{aligned}$$

13 $$\begin{aligned} L_{Dice}(P_{t},L_{S}) = 1-\frac{2\langle L_{Si},P_{ti}\rangle }{\Vert L_{Si}\Vert _{1} + \Vert P_{ti}\Vert _{1}} \end{aligned}$$

For two multiscale feature maps ($P_{t4} = G_{4}(X_{s})$, $P_{t3} = G_{3}(X_{s})$) generated by the segmentation network, their loss function can be defined as:

14 $$\begin{aligned} \begin{aligned} L_{seg4}&= \sigma _{1}(-\beta L_{si}\log P_{t4i} - (1-\beta )(1-L_{Si})\log (1-P_{t4i})) \\&+ \sigma _{2}(1-\frac{2\langle L_{Si},P_{t4i}\rangle }{\Vert L_{Si}\Vert _{1} + \Vert P_{t4i}\Vert _{1}}) \end{aligned} \end{aligned}$$

15 $$\begin{aligned} \begin{aligned} L_{seg3}&= \sigma _{1}(-\beta L_{si}\log P_{t3i} - (1-\beta )(1-L_{Si})\log (1-P_{t3i})) \\&+ \sigma _{2}(1-\frac{2\langle L_{Si},P_{t3i}\rangle }{\Vert L_{Si}\Vert _{1} + \Vert P_{t3i}\Vert _{1}}) \end{aligned} \end{aligned}$$

where $\sigma _{1}$ and $\sigma _{2}$ are the weighted factors to balance the significance of Dice loss and binary cross-entropy loss. $\beta$ is a weighting coefficient to weight the pixel-level positive samples, which makes the network concentrate more on the loss of foreground regions, consequently reducing false negatives. $\langle L_{Si},P_{t3i}\rangle$ and $\Vert L_{Si}\Vert _{1}$ are the dot product and absolute value of the vector matrix, respectively.

##### Adversarial Loss in D1 and D2

The real label images of both inputs $D_{1}$ and $D_{2}$ are $L_{S} = \{L_{Si}, i = 1...n\}$, $L_{Si} =1$ indicating a hippocampus for voxel *i*, while $L_{Si} = 0$ is nonhippocampus. $D_{1}$ and $D_{2}$ play a mini-max two-player game with *G*, and their adversarial loss can be optimized as follows:

16 $$\begin{aligned} L_{adv1} = E_{z\sim L_{S(z)}} [\log D_{1}(z)] + E_{y\sim P_{t4(y)}} [\log D_{1}(G_{4}(y))] \end{aligned}$$

17 $$\begin{aligned} L_{adv2} = E_{z\sim L_{S(z)}} [\log D_{2}(z)] + E_{y\sim P_{t3(y)}} [\log D_{2}(G_{3}(y))] \end{aligned}$$

where $z\in L_{S}$ is the spatial distribution of real label voxels, and *D*(*z*) is the probability of a real image input to $D_{1}$. $y \in P_{t4}$ is the spatial distribution of segmentation feature voxels, and *D*(*G*(*y*)) is the corresponding probability of the prediction feature map. The objective of *G* is to minimize $E_{y\sim P_{t4}(y)}[\log D1(G_{4}(y))]$ and $E_{y\sim P_{t3(y)}} [\log D_{2}(G_{3}(y))$, while $D_{1}$ and $D_{2}$ are to maximize $L_{adv1}$ and $L_{adv2}$,respectively.

##### Information Constraint Loss in $I_{fc}$

The overall optimization goal of training the antagonistic generative network can be formulated as:

18 $$\begin{aligned} \begin{aligned}&FEM,FCM,D_{1},D_{2} = \\&\ \ \ \ \ \arg \min _{(FEM,FCM)}\max _{(D_{1},D_{2})}(\lambda _{1}L_{seg4}+\lambda _{2}L_{seg3}+\gamma _{1}L_{adv1}+\gamma _{2}L_{adv2}), \\&s.t. \ \ \ \ \ \ \ \ \ \ \lambda _{1}+\lambda _{2}+\gamma _{1}+\gamma _{2}=1;\\&\ \ \ \ \ \ \ \ \ \ \ \ \ \ E_{x\sim p(X_{S})} (CL[p(y\mid x)\parallel q(y)]) \le I_{C}\\&\ \ \ \ \ \ \ \ \ \ \ \ \ \ E_{y\sim p(P_{t})} (CL[p(z\mid y)\parallel q(z)]) \le I_{C}\\ \end{aligned} \end{aligned}$$

where $$x\in X_{S}$$, $$y\in P_{t}$$, $$z\in L_{S}$$.

According to mutual information in the information bottleneck, we can calculate the mutual information of the generated prediction map and the ground-truth label image under the network parameter $\zeta$, denoted as:

19 $$\begin{aligned} \begin{aligned}&I(P_{t},L_{s};\xi ) = \iint dy dz p(y,z\mid \zeta ) \log \frac{p(y,z\mid \zeta )}{p(y\mid \zeta )p(z\mid \zeta )} \\&I(P_{t},L_{s};\xi ) \le I_{C} \end{aligned} \end{aligned}$$

$z\in L_{S}$, $y\in P_{t}$, $FEM = p(y\mid z)$ is the probability density of prediction $P_{t}$ under input image $L_{s}$. Accordingly, $p(y\mid \zeta )$ is the probability density of *y* under parameter $\zeta$, and $p(z\mid \zeta )$ is the probability density of *z* under it. Neither can be computed explicitly, so we use $I_{C}$ to constrain their upper bound. For this purpose, we assume that the predicted data (*y*) follow a Gaussian distribution (*q*(*y*)); therefore, the above equation equals:

20 $$\begin{aligned} \begin{aligned} I(P_{t},L_{s};\xi )&= \iint dy dz \ p(y,z\mid \zeta ) p(z) \log \frac{p(y,z\mid \zeta )}{q(y\mid \zeta )} \\&+ \int dy \ p(y\mid \zeta ) \log \frac{q(y\mid \zeta )}{p(y\mid \zeta )},\\ I(P_{t},L_{s};\xi )&\le \int p(z)CL[p(y\mid z)\parallel p(z)]dz \\&= E_{z\sim p(z)}CL[p(y\mid z)\parallel q(y)] \end{aligned} \end{aligned}$$

Similarly,

21 $$\begin{aligned} I(P_{t},X_{s};\xi ) \le E_{x\sim p(x)}CL[p(y\mid x)\parallel q(y)], \ \ x \in X_{S} \end{aligned}$$

Therefore, we impose the *CL* distance as the upper threshold $I_{c}$, so that the noise irrelevant to the segmentation can be explicitly removed from the predicted values. Above all, the information-constrained loss to the inputs ($L_{ic}^{X_{s}}$) can be denoted as:

22 $$\begin{aligned} \begin{aligned} L_{ic}^{X_{s}}&= E_{X_{S}\sim P_{t}} \\&= E_{x\sim FEM(P_{t}\mid X_{S})}(CL[FEM(y\mid x)\parallel q(y)]) - I_{C},\\&\ \ \ \ \ \ \ \ \ \ \ \ \ \ \ \ \ \ \ \ \ \ \ \ \ \ x\in X_{S}, y\in P_{t} \end{aligned} \end{aligned}$$

##### Total Loss Function

Above all, the total loss for our dual constrained adversarial generative network can be expressed as:

23 $$\begin{aligned} \begin{aligned} L_{total}(_{FEM,I_{fc},D_{1},D_{2}})&\\&= \lambda L_{seg} + \gamma L_{adv} + \beta L_{Ic}\\&= \lambda _{1}L_{seg4} + \lambda _{2}L_{seg3} + \gamma _{1} L_{adv1} + \gamma _{2} L_{adv2} + \beta L_{Ic} \end{aligned} \end{aligned}$$

### Postprocessing

To further minimize the background interference effect, we also make two improvements to the posterior probabilities. First, connected component analysis (CCA) is performed. Since our segmentation network is based on 3D voxel decisions, the predictions may contain several noncontiguous areas, for which we set the foreground threshold to 27 (3$\times$3$\times$3). In addition, in type judgment upon each voxel $T_{i}$, for the anterior probability $P_{ant}$, the posterior probability $P_{pos}$, and the background probability $P_{bac}$, we stipulate that

24 $$\begin{aligned} \begin{aligned} T_{i}&= \arg \max _{P_{ant}}, P_{ant} + P_{pos} > 0.5\\&= P_{bac}, P_{ant} + P_{pos} <= 0.5 \end{aligned} \end{aligned}$$

This means that when the probability sum of the anterior and posterior hippocampal tracts exceeds 0.5, we determine it to be the corresponding type of maximum probability; if the background probability exceeds 0.5, we determine it as background directly.

## Results

### Dataset

#### MSD Dataset

**Fig. 7:**
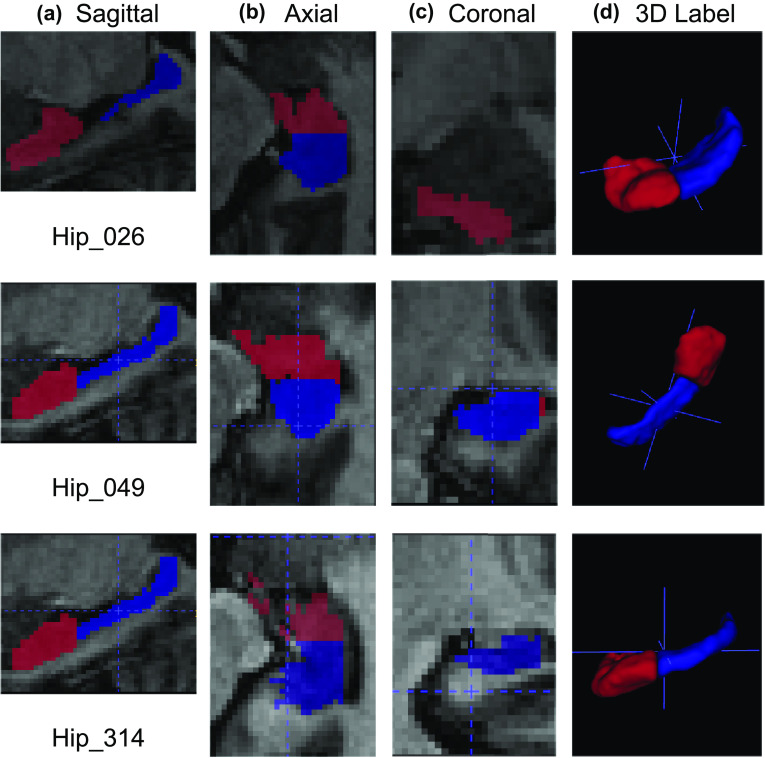
Samples labeled hippocampus in the MSD-H dataset. The red and blue areas indicate the anterior and posterior of the hippocampus, respectively. From left to right in each column: **a** sagittal, **b** axial, **c** coronal, and **d** 3D label

Our methods are mainly evaluated on the MSD dataset published by MICCAI (Medical Image Computing and Computer Assisted Intervention) in 2018. Examples of the Task04 hippocampus dataset (MSD-H) in MSD are shown in Fig. 7. MSD aims to explore multiple anatomies of interest in medical image segmentation models with sophisticated data representation abilities. The competition provides and formats a total of 2,633 3D images for ten popular segmentation tasks: brain tumor (MRI), heart (MRI), liver (CT), hippocampus (MRI), prostate (MRI), lung (CT), pancreas (CT), hepatic vessel (CT), spleen (CT) and colon (CT). The above datasets can be trained individually, while all testing labels are not provided and can only be calculated by the online evaluation platform. However, it measures the ability of a model to segment ten datasets simultaneously and can only be submitted once a day. Therefore, in this paper, we only use the training set of MSD-H (MSD-HT) and apply a tenfold cross-validation strategy to our method.

#### Subjects and Clinical Criteria

MSD-H contains 3D T1-weighted MRIs of the left and right hippocampus of 195 subjects with a total of 390 scans, as detailed in Table 2 [54]. It was provided by Vanderbilt University Medical Center (Nashville, TN, USA) and taken from their Psychiatric Genotype/Phenotype Project data repository. Among them, 90 healthy controls were recruited from the surrounding community. An additional 105 had psychotic disorders (56 cases of schizophrenia, 32 cases of schizoaffective disorder and 17 cases of schizophreniform disorder) from the Vanderbilt Psychotic Disorders Program.

The clinical criteria used here is the Structured Clinical Interview for DSM-IV [55]. Schizophrenia is a splitting of mind, mainly in thought, speech and behavior. Schizoaffective disorder occurs primarily as an affective illness where major depressive, manic or mixed phases play an essential role. Schizophreniform disorder affects shorter time periods (greater than 1 month but less than 6 months) and is less significant than schizophrenia in social functioning. The above-selected patients differ in symptoms but are all subtypes of schizophrenia, and their critical anatomic component is significant atrophy and deformation in their hippocampus.

**Table 2:** Subject demographics and image size information [54]

Subjects	N	Age, years (Mean ±SD)	Gender (Female/Male)	Race (White/Black/Other)	W (min, max)	H (min, max)	D (min, max)
Psychosis	105	34.62 ± 12.38	37/68	63/37/5	31/43	40/59	24/47
Control	90	33 ± 11.33	41/49	60/26/4			

**Fig. 8:**
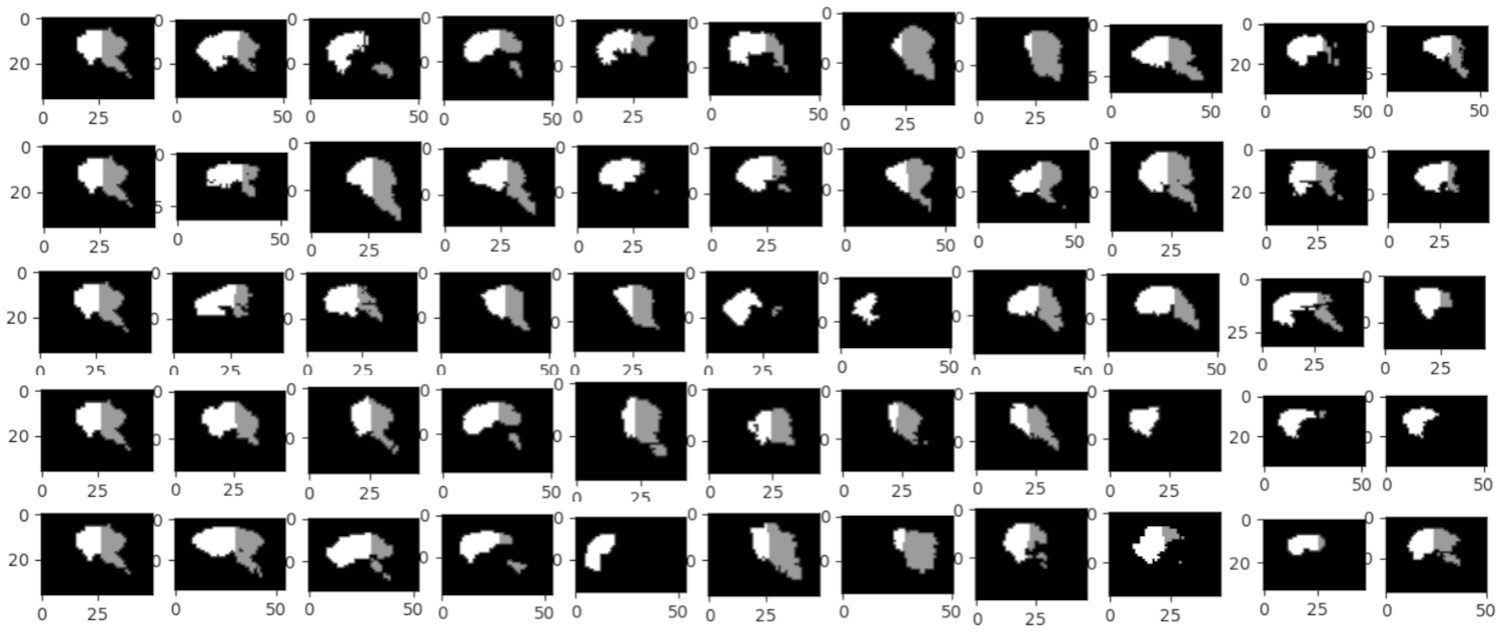
Fifty-five original labels were randomly selected for grayscale displays before preprocessing. For convenient 2D presentation, all images are shown uniformly for the 16th slice. The white area is the anterior of the hippocampus, the gray area is the posterior, and the black area is the background

The goal of this task was to “segment two neighboring small structures with high precision”. All collected data were processed uniformly to the same side, concentrating on the hippocampal region rather than the whole brain, as shown in Fig. 8. The mixed training of all data without distinguishing patients and controls facilitates the validation of the robustness to arbitrary morphological hippocampal segmentation. In addition, data from the left and right hippocampus of the same subject are considered uncorrelated, as they are neighboring but not intersecting at all and have separate corresponding labels. Moreover, the same regions except hippocampal regions of left and right hippocampal imaging in the same subject serve as strong interfering background voxels, which helps to validate the model’s background suppression ability. Furthermore, since the training set contains only 260 of all 390 scans, not all subjects’ left and right hippocampi are included here.

#### MRI Acquisition Parameters

The 390 images in the MSD-H were all 3D T1-weighted MRI acquired by a Philips Achieva scanner, measured by Magnetizeation Prepared-RApid Gradient Echo imaging (MPRAGE) sequence of hippocampal volume (*TI*/*TR*/*TE*=860/8.0/3.7*ms*; 170 *sagittal* *slices*; *voxel* *size*=$1.0 mm^3$). The subject demographics of MSD-H are presented in Table 2. The head (Anterior, $label=1$), body and tail (combined as posterior, $label=2$) of the hippocampus were manually traced in the images following a previously published segmentation scheme [56, 57]. All images were reconstructed from DICOM into Neuroimaging Informatics Technology Initiative (NIfTI) images. As a binary classification task, each image is segmented into two distinguished regions: hippocampal ($label>0$) and nonhippocampal regions ($label=0$).

### Setup

We developed our constructed segmentation algorithm on the Python platform and trained it with four NVIDIA RTX 2080Ti GPUs. To evaluate the performance of the proposed method, we trained 150 epochs with a tenfold cross-validation scheme. By dividing the test set separately in ablation experiments, we eventually chose the mean performance of the tenfold cross-validation as the final model performance. We normalized the data by the *N*(0, 1) normalization method and randomly generated the network weights through a normal distribution. The mean we set was 0, and the mean square error was $\sqrt{\frac{2}{N}}$, where *N* was the outcome number of the previous network layer. Two discriminators were optimized by the stochastic gradient descent (SGD) algorithm, while the generated network leveraged the Nadam optimizer to constrain the learning rate, thus better approaching the optimal prediction performance. More specific hyperparameter configurations are shown in Table 3. Our visualizations were implemented on the ITK-SNAP [58] tool.

**Table 3:** Suggested setting of hyperparameters in the network

**Stage**	**Hyperparameters**	**Value**
Initialization	weight	1.0
	bias	0.0
	power	0.9
	max_iter	500
	learning_rate	2.5E-4
Total Loss	$\sigma _{1}$ of $L_{BCE}$	0.6
	$\sigma _{2}$ of $L_{Dice}$	0.4
	$\lambda _{1}$ of $L_{seg4}$	1
	$\lambda _{2}$ of $L_{seg3}$	0.2
	$\gamma _{1}$ of $L_{adv1}$	0.005
	$\gamma _{2}$ of $L_{adv2}$	0.002
	$\beta$ of $L_{Ic}$	0.001
Training	use_gpu	True
	is_Train	True
	batch_size	16
	max_epoch	151
	momentum	0.9
	rate_decay_weight	$(1-\frac{iter}{max_iter})power$
	GPU RTX 2080Ti	4
Postprocessing	threshold_CCA	27

### Evaluation Metrics

To evaluate the segmentation performance, we take three indicators for significance analysis: Dice similarity coefficient (*DSC*), positive predictive value (*PPV*), and sensitivity (*SEN*).

As a pixel-level segmentation, each voxel is segmented 0 (nonhippocampal pixel) or 1 (hippocampal pixel). We use $GT_{1}$, $GT_{0}$, $PD_{1}$ and $PD_{0}$ to represent the hippocampus in the ground truth (GT), the background region in the GT, the predicted hippocampus region and the predicted background region, respectively. To guarantee that all the above indicators are meaningful, we add a nonzero extreme minimal value ($smooth=0.00001$).

*DSC* measures the degree of overlap between $PD_{1}$ and $GT_{1}$, and the higher the *DSC* value is, the better the segmentation performance. Furthermore, *SEN* visually depicts the probability of $PD_{1}$ in $GT_{1}$, namely, the true positive rate (*TPR*). In other words, the higher the *SEN*, the larger the hippocampal area correctly segmented. *PPV* indicates the probability of $GT_{1}$ in $PD_{1}$, which is the detection rate. The higher the *PPV*, the less background is incorrectly divided into the hippocampus, which means a stronger resistance of the model to the background.

### Ablation Studies

To investigate the high-efficiency training strategy along with the contribution of each key component of DMCA-GAN, we conducted ablation experiments for preprocessing operations, architectural composition, and cross-validation strategies.

#### Comparisons of 3D Patches

In this study, to save hardware resources and speed up the training process, we use the 3D U-Net backbone instead of DMCA-GAN to discover the performance with different patch volumes.

According to Table 2, all voxels range from 31 to 43 (width), 40 to 59 (height), and 24 to 47 (depth). Considering that the average is $35\times 49\times 35$ and the maximum is $43\times 59\times 47$, we properly crop and zero-patch several 3D patches that force at least 1/3 of the samples in each batch to contain prospects. In particular, we compare the performance of three patches $32\times 48\times 32$, $48\times 64\times 48$ and $64\times 96\times 64$. More specifically, to exclude the potential positive bias due to repetitions among samples, we set the same random seeds to generate random batch-size patches for each training batch. As a result, different epochs obtain distinct inputs during training, which also helps the generalization.

**Table 4:** Effect of different patch volumes in hippocampal segmentation with 3D U-Net

Patch size	DSC			PPV	SEN
	Label=1	Label=2	Average		
$32\times 48\times 32$	0.726	0.822	0.774	0.762	0.787
$48\times 64\times 48$	0.830	0.817	0.823	0.879	0.872
$64\times 96\times 64$	0.684	0.768	0.726	0.756	0.762

As shown in Table 4, the worst result is the $64\times 96\times 64$ patch, which means that feeding the entire 3D MR image directly into the training will undoubtedly suffer various background interference issues. When cropping images to $48\times 64\times 48$, all results improved, which confirms the significance of reducing redundant regions for false positive reduction. After proper preprocessing, even the basic U-Net can reach satisfying results. However, when the patch is $32\times 48\times 32$, the anterior DSC, average DSC, and SEN performance decrease by 14.33%, 6.33%, and 10.8%, respectively. More seriously, PPV decreases by 15.35%. This proves that excessive cropping will cause information damage to foreground voxels. As the model trains further, large-size overfitting, as well as small-size contextual information missing, will undoubtedly intensify. Therefore, we set all patches to $48\times 64\times 48$ below.

#### Evaluation of Different Compositions in DMCA-GAN

**Fig. 9:**
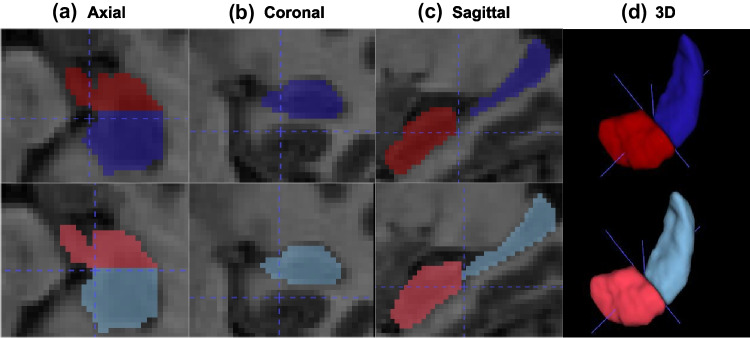
The visualization of DMCA-GAN. The first row shows the label for the axial, coronal, sagittal and 3D levels. The second row shows our corresponding segmentation

To prove the effectiveness of each composition in our DMCA-GAM, we conduct three ablation experiments. First, we compare three basic networks as backbones: *SNG*, $SNG_D1$ and the proposed dual-GAN (baseline). Then, we demonstrate the performance of the proposed *ICL* and *MFCM* on the above three baselines. Finally, a visualization analysis of the optimal network performance is conducted. The specific results are reported in Table 5, and the visualizations are shown in Fig. 9.

**Table 5:** Comparison of the effectiveness with different network compositions

Model	Method	*DSC*			*PPV*	*SEN*
		Label=1	Label=2	Average		
*SNG*	$U-Net$	0.830	0.817	0.823	0.879	0.872
$SNG\_D1$	$U-Net+D1$	0.870	0.857	0.863	0.865	0.892
*Baseline*	$U-Net+D1+D2$	0.877	0.866	0.872	0.909	0.893
*Bottleneck*	$SNG+Bottleneck$	0.851	0.845	0.848	0.830	0.804
$SNG_{ICL}$	$SNG+ICL$	0.864	0.860	0.862	0.825	0.919
$SNG_{ICL}\_D1$	$SNG+ICL+D1$	0.877	0.866	0.872	0.909	0.893
$SNG\_D1_{ICL}$	$SNG+D1+ICL$	0.868	0.849	0.858	0.868	0.890
$Baseline_{ICL}$	$Baseline+ICL$	0.871	0.881	0.876	0.940	0.839
$SNG_{MFCM}$	$SNG+MFCM$	0.876	0.860	0.868	0.830	0.810
$SNG_{MFCM}\_D1$	$SNG+MFCM+D1$	0.886	0.872	0.879	0.967	0.982
$SNG\_D1_{MFCM}$	$SNG+D1+MFCM$	0.882	0.868	0.876	0.981	0.985
$Baseline_{MFCM}$	$Baseline+MFCM$	0.906	0.895	0.900	0.973	0.978
*Proposed*	$DMCA-GAN$	0.911	0.899	0.905	0.967	0.986

##### Results of Network Selection

Among the three benchmarks, the dual-GAN performs optimally, with an average DSC that exceeds that of *SNG* by 5.59% and that of $SNG\_D1$ by 11.04%. These significant improvements demonstrate that the joint adversarial loss of the designed dual-GAN contributes to obtaining superior information. As reported in Table 5, $SNG_{ICL}$ $SNG_{ICL}\_D1$ $SNG\_D1_{ICL}$ $Baseline_{ICL}$ denotes introducing *ICL* unit to the bottom output of *SNG*, the bottom output of *SNG* in $SNG\_D1$, *D*1 output of $SNG\_D1$ as well as both *D*1 and *D*2 outputs of Baseline. As we can observe, compared with their corresponding baselines, $SNG_{ICL}$ $SNG_{ICL}\_D1$ shows excellent improvement, while $SNG\_D1_{ICL}$ and $Baseline_{ICL}$ decrease slightly. This is consistent with the fact that only adding $I_{fc}$ in the *FEM* can effectively suppress the learning of irrelevant noise, thus alleviating overfitting. By the same ablation strategy, the effectiveness of the *MFCM* is manifested by the results among $SNG_{MFCM}$ $SNG_{MFCM}\_D1$ $SNG\_D1_{MFCM}$ and $Baseline_{MFCM}$.

##### Combination with Best Performance

The above detailed comparisons indicate that multiscale feature weighted combined benefits coarse and detailed information learning. Specifically, shallow feature map is similar to the input, and it can nicely capture fine-grained information such as texture and boundaries of the overall hippocampus. However, it tends to contain a lot of noise as well. In contrast, the deep feature map extracted by the network has increased receptive fields and more abstract semantic information such as segmentation specific to each pixel. But it has low resolution and poor perception of details. Extracting and fusing feature maps under different receptive fields for adversarial training can nicely replenish the missing spatial contour details information.

Therefore, we add the *MFCM* at the end of the *FEM* in the generator, applying it at the last two decoding layers of the *FEM*. Moreover, the $I_{fc}$ is introduced to the *FEM* as well, which enables a large increase in both precision and robustness. Our final DMCA-GAN gains the best performance in both *DSC* and *SEN*, which proves its overall segmentation effect and high true positives. The visualization of DMCA-GAN is shown in Fig. 9.

#### Evaluation of Bias Field Correction

To maximally fit the bias field and reconstruct image quality, we first grayscale the image and subsequently attempt five intensity tuning strategies.

**Fig. 10:**
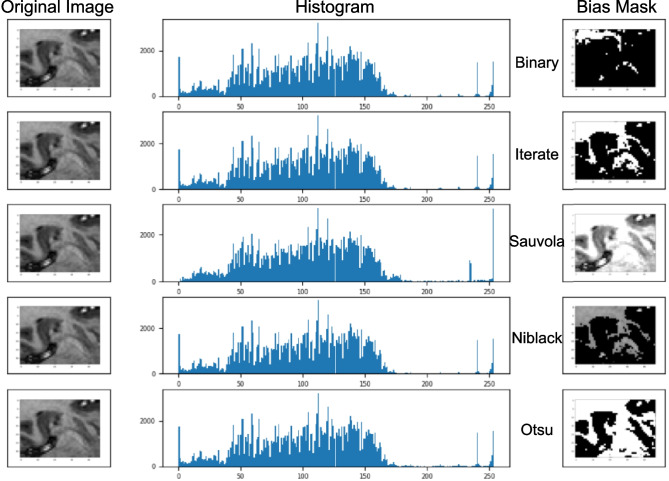
An example of the five correction methods above, from left to right in each line: original image, image histogram after the current correction method, and the corrected result

**Fig. 11:**
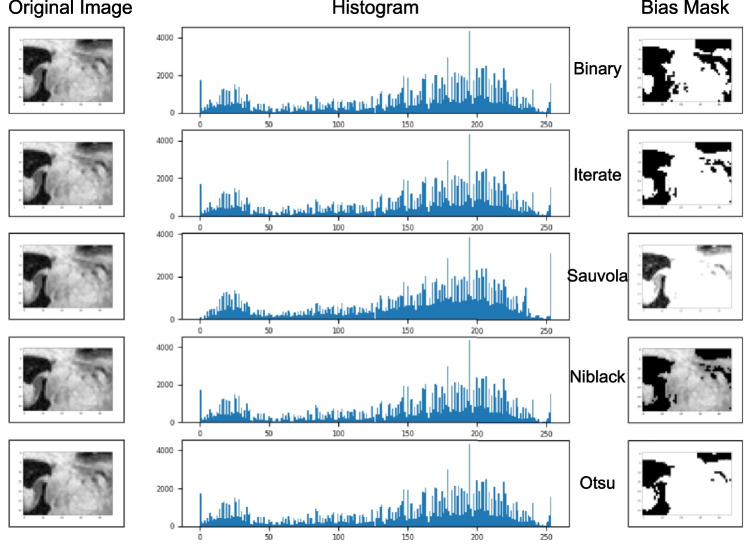
Another example of the comparison among five bias field correction approaches for verifying correction effects

First, binary threshold truncation is performed with the average value of pixels as the threshold. Second, threshold truncation is performed, which is iteratively performed until the average is constant. The following are Sauvola and Niblackboth, both of which are local fields of $16\times 16\times 16$. The final algorithm is Otsu, which traverses all possible thresholds until the maximized interclass variance between foreground and background is found. The correction results are illustrated in Figs. 10 and 11.

**Fig. 12:**
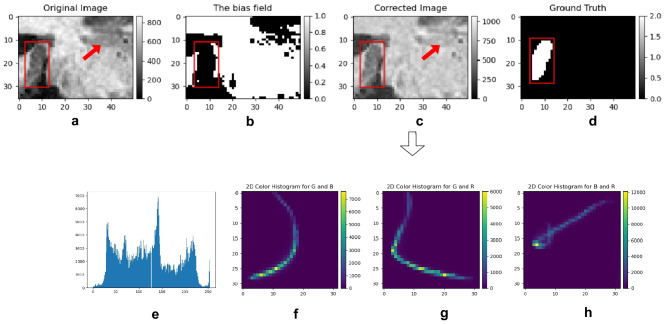
The bias field correction effect of the Otsu algorithm. For ease of observation, the red rectangle shows the hippocampus, and the red arrow represents the significant improvement. (a) Original uncorrected image, (b) bias field calculated by Otsu, (c) corrected image, (d) corresponding labels, (e) corrected 3D image intensity histogram, (f) axial 2D intensity histogram, (g) coronal 2D intensity histogram, and (h) sagittal 2D intensity histogram

As shown in Figs. 10 and 11, globally threshold-based processing works worst, including binary and iterate. It is obviously impossible for complex medical scans to rely on a single threshold for bias field correction. As a local threshold operator, Sauvola performs well in high-contrast images such as Fig. 10 while losing local boundary details in Fig. 11. Since the local histogram peaks in Fig. 11 are not unique, Sauvola exacerbates image blurring instead and loses the details of the target domain. Similar to the local concept of Sauvolaj, Niblacky is also susceptible to local windows and random minima factors, which leads to inconsistent effects.

Additionally, Otsu is less affected by image intensity and contrast, so it can better separate detailed foreground information. Nevertheless, as observed in Figs. 10 and 12, to a certain extent, unrelated regions with similar intensity to the hippocampus are also isolated, which can lead to false positives in subsequent training. In the future, we will further explore robust bias field correction methods to maximize intensity variance and filter out irrelevant background before training.

#### K-Fold Cross-validation analysis

To obtain as much effective information as possible in the limited data learning and considering that there is no category imbalance in the hippocampal segmentation task, that is, all subjects have both anterior and posterior hippocampal tracts, we choose to employ K-fold cross-validation. Each round calculates the mean and standard deviation of the performance on the current model. Finally, the mean and standard deviation of all K-fold scores are taken as the best model generalization hyperparameters.

**Fig. 13:**
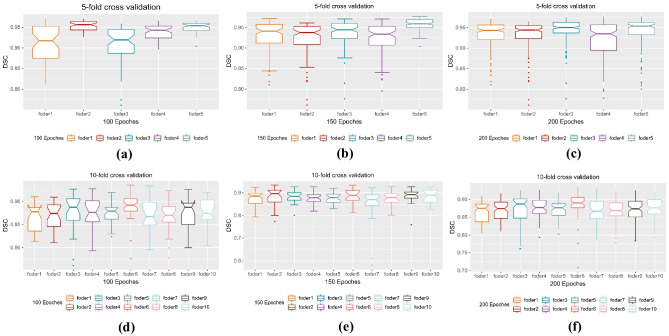
Boxplots of ablation strategies in DSC metrics

We first compared the performance of five times fivefold and ten times tenfold cross-validation on the whole dataset. Figure 13 displays a DSC distribution of per-fold for validation samples, where tenfold cross-validation gains the highest DSC and the most consistent distribution with 150 training epochs. Consequently, we choose it as our optimal training strategy.

To test whether there is overfitting of the optimal strategy, we randomly divide 10% of the training set for testing and rerun ten times tenfold cross-validation, as shown in Table 6. We can see that although a 10% decrease in the training set inevitably leads to a lower *DSC*, its *PPV* and *SEN* are almost unchanged, which indicates that both the detection rate of the foreground and the resistance of the background are not reduced, thereby demonstrating the effectiveness robustness of DMCA-GAN.

**Table 6:** Comparison of K-fold cross-validation

k-fold	epoch	DSC			PPV	SEN
		Label=1	Label=2	Average		
5-fold	100	0.853	0.820	0.836	0.892	0.817
	150	0.885	0.871	0.898	0.918	0.933
10-fold	100	0.887	0.885	0.886	0.882	0.948
	150	0.911	0.899	0.905	0.967	0.986
Test	100	0.806	0.859	0.833	0.887	0.863
	150	0.858	0.832	0.845	0.923	0.910

**Fig. 14:**
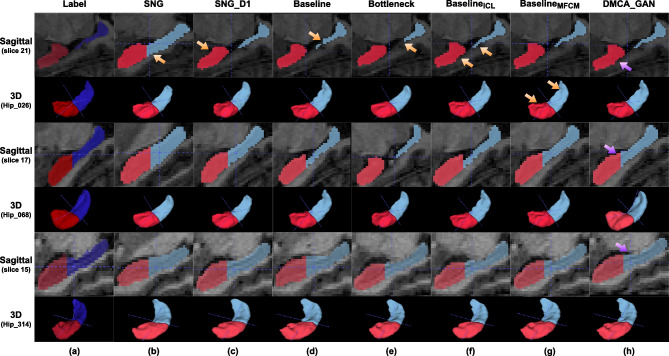
Visualization of sagittal and three-dimensional effects of ablation experiments. The red and blue areas show the anterior and posterior parts of the hippocampus, respectively. The arrows indicate some minor flaws in each ablation model

#### Statistical Significance Analysis of the Results

##### Visual Result Analysis

Figure 14 shows some visual results of ablation studies, with arrows pointing out some minor flaws in each model. It shows that *SNG* suffers severe overfitting. Additionally, although $SNG_D1$ reduces the overall false positives, there is no significant improvement on the anterior area. In addition, our baseline solves this problem better by relying on *D*1 and *D*2. However, due to the strong constraint of the dual discriminator, there are some foreground voxels missing from the posterior. The bottleneck enhances this missing information, while our $I_{fc}$ constrains the effect of background noise better and preserves most of the foreground voxels. In addition, $Baseline_{MFCM}$ further improves the boundary segmentation of $Baseline_{ICL}$, which is not sufficiently smooth. Finally, there is no significant error in DMCA-GAN, which proves the good performance of our model for both anterior and posterior segmentation. Although a certain degree of false positives still exists, certain boundaries are not smooth. Considering the high overall performance of our DMCA-GAN, its overall segmentation effect can be considered reliable and has potential clinical application value.

##### Convergence Analysis

**Fig. 15:**
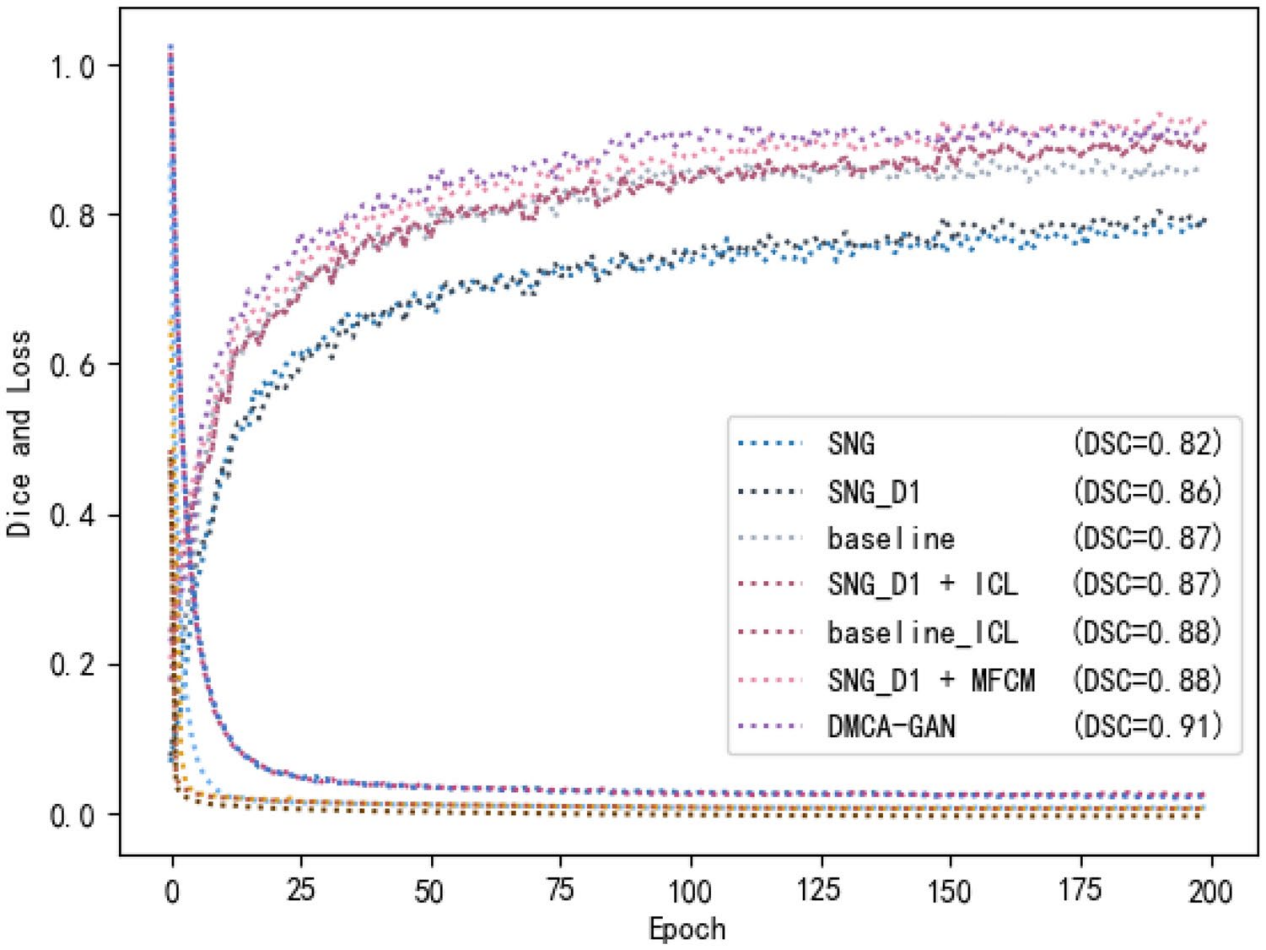
Comparison of the Dice and Loss curves

Figure 15 shows the Dice and loss curves of the above ablation studies. As can be seen, the overall curve tends to be smooth and convergent, while DMCA-GAN performs significantly better than the others. It proves that our proposal can improve the segment performance, as well as resist interference in extreme cases. In the first 30 epochs, all curves change rapidly following a gradient-correlated relationship. Then, all curves level off with Dice increasing to approximately 0.9 and loss dropping to approximately 0.001. At approximately the 150th generation, the Dice stabilizes, and its corresponding loss also no longer decreases, indicating that the gradient update no longer brings the expected information gain. Therefore, to prevent gradient disappearance and gradient explosion, we trained only 150 rounds.

##### Confusion Matrix

**Fig. 16:**
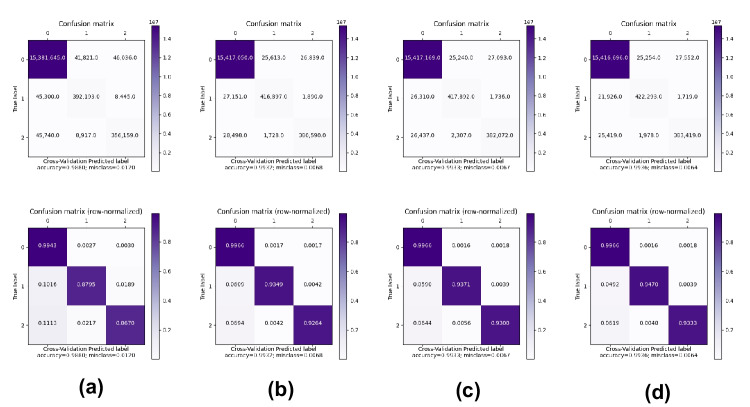
Initial and normalized confusion matrix of four designed networks. (a) $SNG\_D1$, (b) $SNG\_D1_{ICL}$ (c) $SNG\_D1_{MFCM}$ (d) $DMCA\_GAN$

For further comparison of the performance, we also plot the confusion matrix for key components of DMCA. As shown in Fig. 16, to make comparison easier, we further normalize them into the (0, 1) range. It is evident that with the introduction of IFC and MFCN, the prediction accuracy of all models for the three labels has improved substantially.

##### Failure Case Analysis

**Fig. 17:**
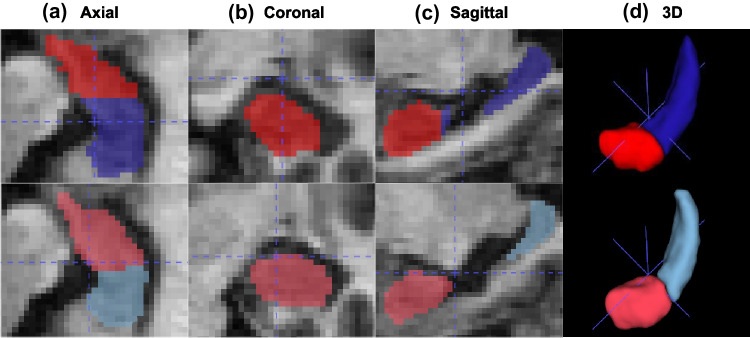
Failure cases of our model. The red and blue areas show the anterior and posterior parts of the hippocampus, respectively. The first row shows the labels for the axial, coronal, sagittal and 3D levels. The second row shows our overfitting segmentation

There are also failure cases where the anterior segmentations tend to be overfitted. As shown in Fig. 17, considering that the MSD dataset contains schizophrenic and healthy control subjects, we suspect that these failure cases may belong to those patients with serious anterior atrophy. A probable reason is that the robustness of our model to handle highly atrophic hippocampus learning is not sufficient.

#### Compare to State-of-art

We compared our result with the state-of-art models. However, since their datasets are different from MSD and some do not provide detailed hyperparameter settings, we cannot obtain results exactly as their original results. For a more objective evaluation, we reproduced them on MSD-H. All the above models use the same hyperparameters, preprocessing, and postprocessing as us. The quantitative results are reported in Table 7.

**Table 7:** Compared with other method results

Model	DSC	PPV	SEN	Year
Modifed nnU-Net [59]	0.78	0.79	0.82	2021
Modifed U-Net [34]	0.88	0.91	0.86	2022
Md-UNet [38]	0.85	0.92	0.85	2020
MDL [60]	0.87	0.95	0.92	2018
CAST [32]	0.89	0.91	0.93	2020
Ours	0.91	0.97	0.99	2023

As we can see from the results, in terms of small size and high noise level in hippocampus segmentation, the semisupervised conditional nnU-Net proposed by Zhang et al. [59] cannot match our technology in all three metrics. As an improvement of the U-Net architecture, Hazarika et al. [34] improved the segmentation accuracy by adding multiple convolutional filters, but it also needed to extract more parameters, resulting in a high computational cost to the model. Moreover, their result was slightly lower than ours. For the unsupervised domain adaptive strategy, Lin et al. [38] designed dual-branch with improved SSA adapters to enhance small-size image segmentation. Cao et al. [60] utilized existing standard templates to normalize and extract image patches and put them into the network for adaptive learning without any additional preprocessing. Both of them improved the DSC and PPV of segmentation, but the SEN was fairly low. This result indicated that there was still a gap between the segmented region and the real hippocampus region. This may be caused by the existence of many outliers. Finally, compared to the multimodel deep convolutional neural network (CAST) proposed by Liu et al. [32], our method is the same as theirs in *PPV* index and has slight advantages in *DSC* and *SEN*. However, there is room for improvement in the robustness and generalization of DMCA-GAN.

## Discussion and Conclusion

In this work, the convolution-based alternating adversarial training process obtained multiscale pixel-level prediction results. Two discriminators we developed effectively combine global positioning information and local refining boundary information, which contributes to refining the segmentation boundaries and preventing the negative migration phenomenon in adversarial training. In addition, the information entropy constraint unit proposed in the FEM of G enhanced the filtering ability of MRI high-frequency noise and thereby prevented overfitting. Finally, we constructed a multilevel feature extraction attention mechanism, which takes advantage of the multiscale weighting strategy to reduce noise weight, thus forcing the network to concentrate more on effective details at various scales. Meanwhile, it strengthened feature propagation and reused underlying features, thus preventing overfitting. In addition, we also explored appropriate 3D patch random sampling and offset field correction strategies, which also contributed to the improvement of segmentation performance.

Our method focused on the exact clinical task of precise hippocampus segmentation in high-frequency MR images, and demonstrated decent segmentation performances on the MSD dataset, specifically in comparison with recently proposed state-of-the-art methods. However, it is insufficient for precise anterior segmentation, which tends to be overfitting. One probable reason is that the robustness of our model to handle highly atrophic hippocampus learning is not enough. Although we have improved the performance with postprocessing to a certain extent, there is still room to improve the segmentation of the atrophic anterior. Considering that hippocampal abnormalities have been demonstrated in schizophrenia or other neuropsychiatric disorders, the precise segmentation of the hippocampus, especially in those with neuropsychiatric disorders would be technically challenging with clinically important implications.

In addition, it still has much room to improve the boundary segmentation performance and reduce false positives. As the study progressed, we realized that excellent preprocessing is even more important than segmentation network construction. For example, more advanced methods for bias field correction may be helpful to improve the performance of the same segmentation approach. Besides, we will also consider multisource image information complementation [61], such as the Deep Label Fusion (DLF) method proposed by Long et al. [62], which combines the strengths of deformable image registration and multiatlas label fusion. In addition, a differentiable topology search of the network [63] to overcome the limitation of traditional U-shaped space may be also a promising development direction. The above will be the direction of our further work.

## Data Availability

The dataset is public and can be downloaded from http://medicaldecathlon.com/.

## Code Availability

The code used in this work is available from the first author upon request.
